# Educational initiative in an NCATS TL1 training program to address the impact of systemic racism on human health, biomedical research, and the translational scientist

**DOI:** 10.1017/cts.2022.500

**Published:** 2022-11-14

**Authors:** Martha D. Gay, Kimberly A. Bell, Emily A. Bujold, Marilla Geraci, Dexter L. Lee, Kathryn Sandberg, Robert C. Speth

**Affiliations:** 1 Georgetown-Howard Universities, Center for Clinical and Translational Science, Washington, DC, USA; 2 Department of Medicine, Georgetown University, Washington, DC, USA; 3 Department of Psychology, North Carolina Agricultural and Technical State University, Greensboro, NC, USA; 4 Ingenesis, Inc., San Antonio, TX, USA; 5 Department of Physiology, Howard University, Washington, DC, USA; 6 Department of Pharmaceutical Sciences, Nova Southeastern University, Fort Lauderdale, FL USA

**Keywords:** Underrepresented, diversity, CTSA, equity, unconscious bias

## Abstract

**Introduction:**

The goal of clinical and translational science (CTS) is to fill gaps in medical knowledge toward improving human health. However, one of our most pressing challenges does not reside within the biological map we navigate to find sustainable cures but rather the moral compass to recognize and overcome racial and ethnic injustices that continue to influence our society and hinder diverse research rigor. The Georgetown-Howard Universities Center for Clinical and Translational Science includes an inter-institutional TL1-funded training program for predoctoral/postdoctoral trainees in Translational Biomedical Science (TBS).

**Methods:**

In the fall of 2020, the TBS program responded to the national social justice crisis by incorporating a curriculum focused on structural racism in biomedical research. Educational platforms, including movie reviews, Journal Clubs, and other workshops, were threaded throughout the curriculum by ensuring safe spaces to discuss racial and ethnic injustices and providing trainees with practical steps to recognize, approach, and respond to these harmful biases in the CTS. Workshops also focused on why individuals underrepresented in science are vital for addressing and closing gaps in CTS.

**Results:**

Paring analysis using REDCap software de-identified participants after invitations were sent and collected in the system to maintain anonymity for pre- and post-analysis. The Likert scale evaluated respondents’ understanding of diverse scientific circumstances. The pre/Fall and post/Spring surveys suggested this curriculum was successful at raising institutional awareness of racial and ethnic biases. Evaluating the effectiveness of our program with other training Clinical and Translational Science Awards (CTSA) consortiums will strengthen both the academic and professional TBS programs.

## Introduction

The death of George Floyd in the summer of 2020 instigated a wake-up call heard worldwide that racism continues to plague society. Introspection ensued, including within the medical educator community. Directors of training programs revisited their approach to addressing racism and prejudice in biomedical research. Required training in human subject research includes “The Tuskegee Study of Untreated Syphilis in the Negro Male” as the iconic exemplar of abuse of Blacks by the medical profession [1]. However, this study is just one example of how Blacks have been used for medical experimentation dating back hundreds of years [2], when they were considered without rights as human beings [3] and expendable in the service of providing medicine for White people [4,5]. Moreover, as Linda Villarosa succinctly stated, “African Americans live sicker and die quicker” [6]. Thus, it should come as no surprise that distrust of the medical establishment is widespread among Black and other minority populations [7–10]. This distrust contributes to why clinical trials struggle to reach their recruitment goals for diversity and why clinical findings are often not powered to assess outcomes in minority subpopulations [11]. Reducing health disparities in incidence and outcomes continues to be part of the mission of the National Center for Advancing Translational Science (NCATS) at the National Institutes of Health (NIH).

Shortly after the death of George Floyd, Dr. Francis Collins, then Director of the NIH, apologized for longstanding and continuing structural racism in the biomedical sciences. He acknowledged that significant changes at NIH were necessary to effectively address structural racism in biomedical research. Despite all previous efforts to raise awareness of the importance of diversity and inclusivity, the numbers continue to reflect a large discrepancy between Whites and minorities in grant applications, funding rates, and numbers of postdoctoral fellows, staff, and senior scientists [12]. To this end, together, we must act—and we must act now by being active participants in ending structural racism.

Several NIH institute directors followed Dr. Collins’ lead and developed committees to address structural racism. On February 26, 2021, a special meeting of the advisory committee to the director announced the establishment of the UNITE initiative (launched on March 1, 2021) to bring together all the NIH Institutes and Centers to focus on addressing racism and discrimination in science and advancing the goal of diversity and inclusion throughout the biomedical community through five separate committees including U: Understanding stakeholder experiences through listening and learning; N: New research on health disparities, minority health, and health equity; I: Improving the NIH culture and structure for equity, inclusion, and excellence; T: Transparency, communication, and accountability with our internal and external stakeholders; and E: Extramural research ecosystem: changing policy, culture, and structure to promote workforce diversity [13].

On March 1, 2021, Dr. Joni L. Rutter, then Acting Director of the NCATS, voiced a commitment to join Dr. Collins and “stand alongside the rest of NIH in its commitment to end structural racism in biomedical research” and join the UNITE initiative. Dr. Rutter goes on to say, “At NCATS, we have committed ourselves — in words and in actions — to support diversity, equity and inclusion and to address the scientific, operational, organizational and cultural problems that have contributed to racial inequities across the biomedical research enterprise” [14].

The Georgetown-Howard Universities Center for Clinical and Translational Science (GHUCCTS) community was also shocked by the murder of George Floyd. In direct response to those events, our NCATS-supported TBS training program for predoctoral students and postdoctoral fellows tasked a Program Committee on July 13, 2020, to incorporate a focus on systemic racism in biomedical research throughout the TBS curriculum. Members included the co-directors of the TBS program (DLL and KS), a former TL1 trainee (MDG), the Associate Director (EAB), a biomedical scientist who studies the impact of racism on health (KAB), basic scientists (MG, DLL, KS, and RCS), clinical research investigators (KAB, MG, and MDG), and a bioethicist educator (RCS). This paper describes our experience implementing a pilot program to raise awareness of systemic and structural racism in biomedical research in an early career clinical and translational science (CTS) cohort.

## Methods

### Program Components

The curriculum was integrated into the Translational Biomedical Science (TBS) program between September 2020 and August 2021. The curriculum included movie and Journal Clubs, workshops in the Responsible Conduct of Research (RCR), and Visiting Professor Series throughout the academic year. Additional resources beyond the program’s workshops were provided. The homework included the online Harvard Implicit Association Test, which measures attitudes and beliefs that people may be unwilling or unable to report [15]. Trainees were asked to take the Harvard Implicit Project Test, only at the beginning of the semester. They were tasked to complete 15 surveys within 2 weeks, which approximately took an overall work effort of 90 minutes to finish. No post changes were observed because we did not ask them to confirm or provide any details once they completed it. The learning objective for this exercise was to introduce the scholars to how often they may have experienced implicit biases during everyday interactions.

#### Movie Club

All trainees were required to watch three movies from October 2020 through May 2021. Movies included *Something the Lord Made*, *The Immortal Life of Henrietta Lack*s, and *Miss Evers’ Boys* (Table 1). All films had a component of racial injustice, including the role of ethics in biomedical research and the impact on researchers and participants. Trainees were divided into three groups of three or four trainees each to give a 15-minute presentation and lead a 60-minute discussion. Each group was randomly assigned to one movie and was tasked to meet prior to the Movie Club workshop to develop a short presentation and then lead a productive and non-confrontational discussion around movie-specific questions (Table 2). The Scholar-in-Chief, MDG, was the writer of the questions, with feedback from the Program Committee and she also participated in the movie review groups. These questions provided a platform for galvanizing a productive discussion related to recognizing, addressing, and combatting racism in science.

**Table 1.** Movie Club

Date	Movie (Year)/Writers and Directors
10/29/20	*Something the Lord Made* (2004) Writers: Peter Silverman and Robert Caswell Director: Joseph Sargent
12/10/20	*The Immortal Life of Henrietta Lacks* (2017) Screenplay: Peter Landesman, Alexander Woo, and George C. Wolfe (Based on book by Rebecca Skloot) Director: George C. Wolfe
05/06/21	*Miss Ever’s Boys* (1997) Writer: Walter Bernstein Director: Joseph Sargent

Racial awareness movies. All these movies portray racial injustice in science. Trainees were instructed to use the movies as an evaluation tool to create and led question-based presentations. Certain controversial elements from each movie were integrated into the Responsible Conduct of Research workshops.

**Table 2.** Movie Club Discussion Questions (Q)

*Something the Lord Made*	
Q1	Why do you think Dr. Alfred Blalock gave Mr. Vivien Thomas a white lab coat?
Q2	What were the moments in the movie when Vivien felt like he was Dr. Blalock’s colleague?
Q3	In your opinion, do you think Dr. Blalock was using Vivien, or were they using each other?
Q4	Was the honorary degree and portrait for Mr. Vivien Thomas enough reparations for his contributions to heart surgery, and why were his innovative accomplishments recognized after Dr. Blalock’s death.
Q5	A room full of doctors witnessed Vivien’s involvement in the “Blue Baby” surgery, and although his support saved a life, he was not given the respect he earned. Explain how you think Vivien felt when he was not included and do you think in 2020 would things be different?
Q6	Although Vivien’s title was classified as a janitor when he was a lab tech, do you think a similar pay scale and inaccurate job description still exists with people of Color today?
Q7	How did Vivien Thomas exhort others to respect him and was he successful?
*The Immortal Life of Henrietta Lacks*	
Q1	Does the end – i.e., the immeasurable benefit to humankind resulting from those tissue samples – justify the means – i.e., removing tissue from a person without their consent or knowledge? Explain your position?
Q2	Did socioeconomic status or race impact Henrietta’s treatment?
Q3	Do you have a responsibility to correct prior scientific injustices as you conduct your present/future research?
Q4	How has the implementation of “informed consent” changed since Henrietta’s day in the 1950s and what were some examples shown in the movie of Doctor/Patient misinformation?
Q5	What kinds of innovations have come about because of HeLa cells?
Q6	Do you think Henrietta Lack’s descendants should be compensated for her contribution to science? Why?
Q7	How do you ensure that the human tissue or human cells you are using in your experiments were obtained with appropriate informed consent? What would you do if you discovered they weren’t?
*Miss Evers’ Boys*	
Group 1:	
Q1	In the researchers’ initial proposal, how did they justify their attempt to advance the medical field? Explain what Miss Evers’ initial reason for participating in the study was?
Q2	Why didn’t the “boys” receive penicillin when it became available?
Group 2:	
Q1	Due to the ethical concerns of the study, would you discard all results or publish them?
Q2	Do you think this study would have been done in a similar socioeconomic status in a White community?
Group 3:	
Q1	Did the study accurately determine the fundamental research question: “Is the rate and progression of Syphilis in African American males the same as that for European males?” Explain.
Q2	What do you think about the Tuskegee Experiment? How did this movie make you feel as a researcher?

Racial awareness movies questions. To answer each film’s assigned questions, the trainees were randomly divided into groups. They used the movies as an evaluation tool to create and led question-based presentations for safe space conversations. After the movie review, tools to recognize and respond to biases were discussed.

#### Journal Club

This was a new component integrated into our program’s curriculum to evaluate and discuss scientific papers related to health disparities, which ran from January to May. All trainees were required to read and spearhead the talks from four Journal Club articles. Randomly selected trainees were divided into two groups of 2–3 each and presented their article in-depth for a 20-minute presentation followed by a 60-minute discussion. Each group was tasked to meet prior to the Journal Club workshop and develop a short presentation with questions geared toward leading a discussion around the article(s) presented (Table 3). The facilitators generated these questions to serve as a platform for stimulating a productive conversation related to study findings, interpretation, and critique of the methods as well as experimental limitations, with an additional emphasis placed on the root cause analysis of health disparities.

**Table 3.** Journal Club

Date	Workshop Title/ Speakers/Facilitators	Articles Reviewed
2/4/21	*COVID-19 therapeutics* Guest Speaker: John Kwagyan, PhD Assistant Professor MPH Program and Graduate School HUCM Facilitators: Chaz Hinzman, PhD TL1-PhD Student, GUMC; Collis Brown, PhD TL1-PhD Student, HUCM	Hippisley-Cox J, et al. *Risk of severe COVID-19 disease with ACE inhibitors and angiotensin receptor blocks: cohort study including 8.3 million people* [16]
03/04/21	*Disparities in outcomes with COVID-19* Facilitators: Martha D. Gay, PhD TL1 Scholar-in-Chief, GUMC Joseph Aubee TL1-PhD Student, HUCM Alison Schug TL1-PhD Student, GUMC	Azar, KMA et al. *Disparities in outcomes among COVID-19 patients in a large health care system* [17] Ogedegbe, G et al. *Assessment of racial/ethnic disparities in hospitalization and mortality in patients with COVID-19* [18]

Journal Club articles. Randomly selected trainees from Howard University College of Medicine (HUCM) and Georgetown University Medical Center (GUMC) were divided into two groups to develop a presentation based upon questions by the Facilitators.

#### Seminar workshops

We integrated racial awareness topics into four of our five RCR series that ran from March to June 2021. These workshops included discussions lead by Experts on health disparities and the occurrence of racial and ethnic bias in biomedical research (Table 4). In addition, three workshops in the Visiting Scientist series between October 2020 and April 2021 specifically addressed health disparities in research outcomes and educational approaches to raising awareness of unconscious racial bias (Table 5).

**Table 4.** RCR Series

Date	Speakers	Talk Titles
03/11/21	Kathryn Sandberg, PhD Professor Dept. of Medicine GUMC	*Rigor and reproducibility in the time of COVID*
04/08/21	Mary Young, MD Assistant Professor Dept. of Medicine GUMC	*How to write an effective institutional review board application*
05/06/21	Robert C. Speth, PhD Professor Dept. of Pharmaceutical Sciences Nova Southeastern University	*Informed consent and respect for personhood*
05/27/21	Breakout Room Facilitators: Robert C. Speth, PhD Professor Dept. of Pharmaceutical Sciences Nova Southeastern University Dexter L. Lee, PhD Associate Professor Dept. of Physiology and Biophysics HUCM Kimberly A. Bell, PhD Assistant Professor Dept. of Psychology North Carolina Agricultural and Technical State University	*Scientists as a responsible member of society:* *The Pandemic of 1918* *COVID vaccine long-term effects* *Human Cloning*

Responsible conduct of research (RCR) workshops. The RCR workshops that had Racial Awareness topics were discussed and led by Experts on health disparities and the role of racial and ethnic bias in biomedical research.

**Table 5.** Visiting Professor Series

Date	Speaker	Talk Title
10/08/20	Lisa M. Harrison-Bernard, PhD Professor Dept. of Physiology Louisiana State University School of Medicine	*Understanding diversity, equity, inclusion, and implicit bias (Part I)*
10/08/20	Kimberly A. Bell, PhD Assistant Professor Dept. of Psychology North Carolina Agricultural and Technical State University	*Impact of perceived racism on cardiovascular health*
04/22/21	Lisa M. Harrison-Bernard, PhD Professor Dept. of Physiology Louisiana State University School of Medicine	*Understanding diversity, equity, inclusion, and implicit bias (Part II)*

Visiting Professor Series. The Experts on health disparities and the role of racial and ethnic bias in biomedical research and educational approaches to raising awareness of unconscious and racial bias

### Surveys

Survey statements were written by the Scholar-in-Chief, MDG, and disseminated through REDCap software to capture feelings and opinions using a 5.0 Likert scale (Table 6) using the following five statement points: (1) strongly disagree; (2) disagree; (3) neutral; (4) agree; and (5) strongly agree. This Likert scale was the same for all pre- and post-survey questions. Paring analysis using REDCap software de-identified participants after invitations were sent and collected in the system to maintain anonymity for pre- and post-analysis. Online survey was administered to the TL1 cohort beginning and after the year-long program. Survey participation was optional and 100% of TBS GHUCCTS trainee responses were recorded. The survey cohort consisted of 2 postdoctoral fellows and 10 predoctoral students at GHUCCTS institutions. All results were reported anonymously. Ten participants completed both pre- and post-surveys.

**Table 6.** Pre- and post-survey statements (S)

#	Statements		
S1.	I know what to say when interacting with people from different cultures.		
		Pre	Post
	Mean	3.8	4.1
	Standard error	0.31	
		*P* = 0.034	
S2	I am just as comfortable talking to individuals from different cultures as to those from my own culture.		
		Pre	Post
	Mean	4.0	4.2
	Standard error	0.25	
	*P* = 0.43		
S3	I have awareness of unconscious biases and stereotypes.		
		Pre	Post
	Mean	4	4.6
	Standard error	0.34	
		*P* = 0.09	
S4	I am able to recognize biases in biomedical research, education, and training.		
		Pre	Post
	Mean	3.7	4.4
	Standard error	0.36	
	*P* = 0.06		
S5	I have observed bias in my interactions with mentors and teachers.		
		Pre	Post
	Mean	3.4	3.5
	Standard error	0.49	
	*P* = 0.84		
S6	I have observed bias in my interactions with peers.		
		Pre	Post
	Mean	3.3	3.3
	Standard error	0.44	
	*P < 0.0001		
S7	I have observed bias in my interactions with mentees (e.g., research intern under your supervision).		
		Pre	Post
	Mean	2.6	2.7
	Standard error	0.34	
	*P* = 0.77		
S8	I am aware of the specific obstacles that many individuals underrepresented in biomedical science have encountered.		
		Pre	Post
	Mean	3.2	4.1
	Standard error	0.50	
	*P* = 0.08		
S9	I am confident in my ability to identify individuals from groups underrepresented in the biomedical sciences.		
		Pre	Post
	Mean	3.7	4.0
	Standard error	0.37	
	*P* = 0.43		
S10	Scientists from different cultures improve biomedical research.		
		Pre	Post
	Mean	4.8	4.9
	Standard error	0.22	
	*P* = 0.66		
S11	Do you seek out the perspectives from people of different cultures in your research?		
		Pre	Post
	Mean	3.1	4.0
	Standard error	0.46	
	*P* = 0.06		

Pre- and post-survey statements. Designed in REDCap to capture thoughts and opinions using the Likert scale. The anonymous on-line survey was administered to the TL1 cohort at the beginning and at the conclusion of the year-long program. The mean and standard deviation were calculated. The t-test was applied to calculate the *P*–value, but there was no significant difference between the two groups except for S6 * *P* < 0.05.

## Results

Surveying our TL1 cohort of before and after instituting this educational pilot suggests the trainees gained an appreciation of the negative effects systemic and structural racism have on the biomedical workforce, research, and health care. Furthermore, the demographic information for the 2020–2021 TBS cycle for Georgetown University (GU) and Howard University (HU) TBS participants is as follows: ten predoctoral students which consisted of 8 GU and 2 HU, 5 female and 5 male, 8 White and 2 African American, and 1 Hispanic and 9 Non-Hispanic scholars. Furthermore, the program had two GU postdoctoral fellows, 1 female and 1 male, 1 Withheld and 1 White, respectively, and 2 Non-Hispanic scholars.

**Fig. 1.**
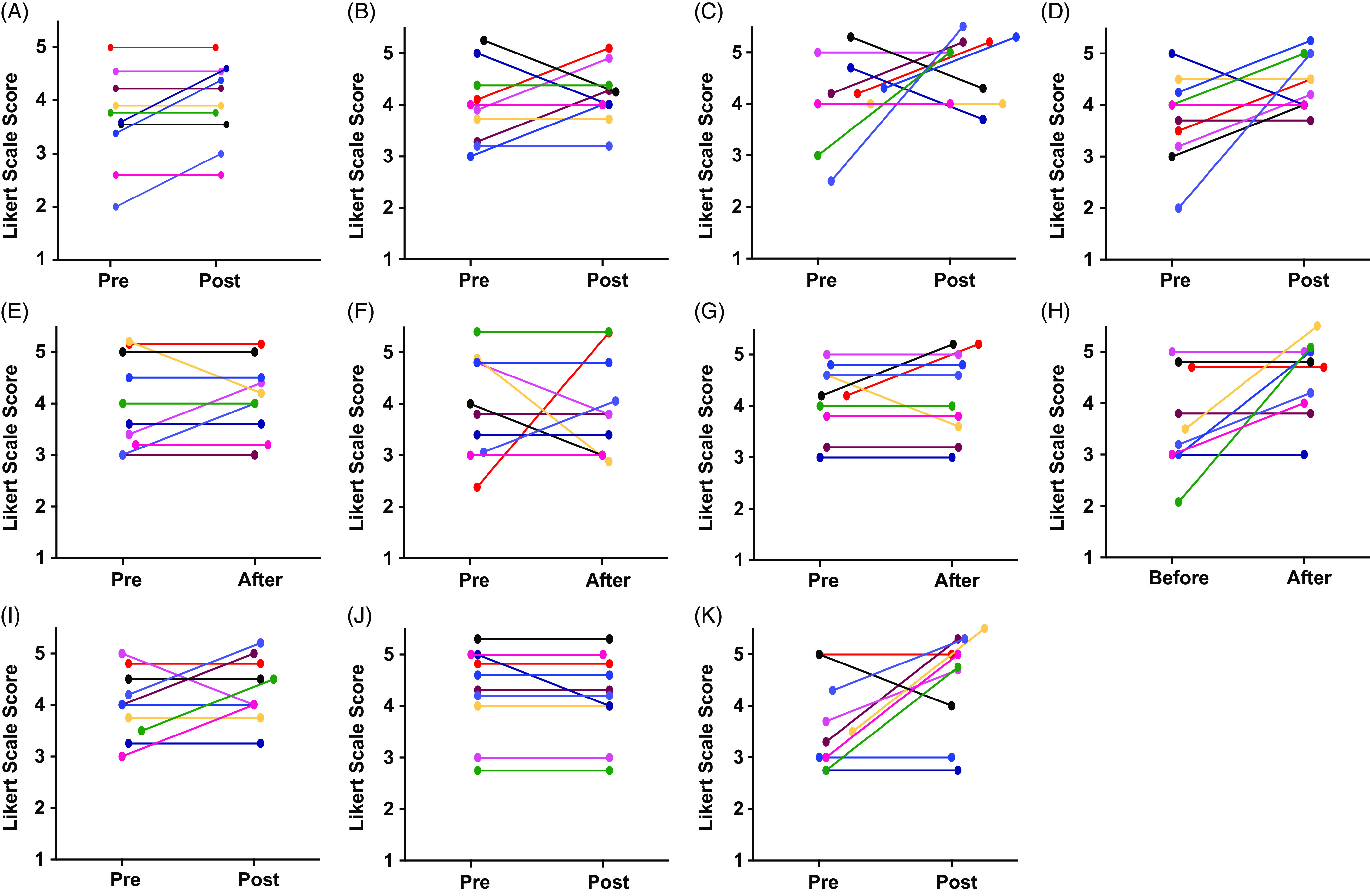
Pre- and post-surveys. Figures (A–K) used the 5-point Likert scale ranging from (1) strongly disagree, (2) disagree, (3) neutral, (4) agree, and (5) strongly agree. The graphs represent individual responses from all ten participants. The anonymous online questionnaire was administered at the beginning and the end of the year-long program. Figures (A–B) evaluate the comfort level of the trainee in conversing amongst diverse researchers other than their ethnic background, and Figures (C–K) were specifically on bias recognition in research.

*S1: I know what to say when interacting with people from different cultures.* Refer to (Fig. 1A), the average initial response was 3.8 on the 5.0 Likert scale, which increased to 4.1 at the end of the program. While the majority of participants did not change their responses, three moved from neutral to agree. No one lowered their agreement.

*S2: I am just as comfortable talking to individuals from different cultures as to those from my own culture.* Refer to Fig. 1B, the average initial response was a 4.0 on the 5.0 Likert scale, which increased to 4.2 at the end of the program. Half of the trainees changed their response, with three moving to a greater agreement, while two moved from strongly agree to agree.

*S3: I have awareness of unconscious biases and stereotypes.* Refer to Fig. 1C, the average initial response was a 4.0 on the 5.0 Likert scale, which increased to 4.6 at the end of the program. Seventy percent of participants changed their response, with half moving to greater agreement including one individual from neutral to strongly agree and another from disagree to strongly agree. Two lowered their agreement moving from strongly agree to agree.

*S4: I am able to recognize biases in biomedical research, education, and training.* Refer to Fig. 1D, the average initial response was a 3.7 on the 5.0 Likert scale, which increased to 4.4 at the end of the program. Seventy percent of participants changed their response. Sixty percent moved to greater agreement with one individual moving from disagree to agree and another moving from strongly agree to agree.

*S5: I have observed bias in my interactions with mentors and teachers.* Refer to Fig. 1E, the average initial response was a 3.4 on the 5.0 Likert scale, which increased by 0.1 making it 3.5 greater by the end of the program. Thirty percent of participants changed their response with 20% moving to greater agreement, while one moved from disagree to strongly disagree.

*S6: I have observed bias in my interactions with peers.* Refer to Fig. 1F to observe, the average initial response was a 3.0 on the 5.0 Likert scale. There was no change in the average response at the end of the program. However, fifty percent of participants changed their response. Twenty percent moved to greater agreement including one individual who went from disagree to strongly agree, while 30% moved to lower agreement including one individual that went from agree to disagree.

*S7: I have observed bias in my interactions with mentees (e.g., research intern under your supervision).* Refer to Fig. 1G to observe, the average initial response was a 2.6 on the 5.0 Likert scale, which increased slightly to 2.7 at the end of the program. However, 30% of participants changed their response. Twenty percent of the participants moved to greater agreement from disagree to neutral, while one individual lowered their response from disagree to strongly disagree.

*S8: I am aware of the specific obstacles that many individuals underrepresented in biomedical sciences have encountered in their careers.* Refer to Fig. 1H to observe, the average initial response was a 3.2 on the 5.0 Likert scale, which increased to 4.1 at the end of the program. Fifty percent of participants changed their response with all moving to greater agreement. Of note four respondents disagreed initially and three of those four moved to agree or strongly agree.

*S9: I am confident in my ability to identify individuals from groups underrepresented in the biomedical sciences.* Refer to Fig. 1I to observe, the average initial response was a 3.7 on the 5.0 Likert scale, which increased to 4.0 at the end of the program. Fifty percent of participants changed their response with 40% moving to greater agreement while one individual moved down from strongly agree to agree.

*S10: Scientists from different cultures improve biomedical research.* Refer to Fig. 1J to observe, the average initial response was a 4.8 on the 5.0 Likert scale, which did not change at the end of the program. Only two individuals changed their response with one individual moving from neutral to agree and the other in the opposite direction from strongly agree to agree.

*S11: Do you seek out the perspectives from people of different cultures in your research?* Refer to Fig. 1K to observe, the average initial response was a 3.1 on the 5.0 Likert scale, which increased to 4.0 at end of the program. Seventy percent changed their response with 60% moving to greater agreement. Four participants increased their degree of agreement by two points, while the other two increased their agreement by one point. Only one individual lowered their agreement from strongly agree to agree.

Statements 1 and 2 addressed the comfort level trainees have interacting with individuals from different cultures. The majority of the trainees expressed positive responses (agree or strongly agree) with knowing what to say to people from different cultures; none disagreed (Fig. 1A). Only a few changed their initial response at the end of the program; of these, all except one moved toward greater positivity. Similarly, when asked about their comfort level in conversing with those from different cultures relative to their own culture, most trainees expressed positive agreement (Fig. 1B). However, half of the trainees changed their response by the end of the program with some moving toward greater agreement while others moved toward lesser agreement. One interpretation of the mixed change in direction between pre- and post-survey responses to this statement is that the greater awareness of how discrimination can lead to major differences in life experiences depending upon one’s race or ethnicity gave some trainees pause when conversing with those from other cultures.

This theme was brought home by the Movie Club workshop on *Something the Lord Made*, which included a discussion of the negative impact of racism on biomedical scientists then and now. This film based on a true story is about Vivien Thomas, a black man in the 1930s during an era of blatant racial injustice in academia. Originally hired as a janitor, Mr. Thomas played an essential role in Dr. Alfred Blalock’s medical research on the “Blue Baby Syndrome.” Although Mr. Thomas was indispensable to Blalock’s progress, he received no credit for his contribution to the groundbreaking therapy, with his job remaining classified as a janitor. Finally, in 1976, Johns Hopkins awarded Mr. Thomas with an honorary Doctorate for his contributions to vascular and cardiac surgery.

All the trainees expressed amazement at Mr. Thomas’ development of a surgical technique to repair the cardiac defect, tetralogy of Fallot (i.e., Blue Baby Syndrome), especially considering minimal mentorship from Blalock. They voiced frustration regarding Blalock’s refusal to support Mr. Thomas’ pursuit of a medical degree and professional advancement, despite Thomas’ qualification for such. They also expressed anger over Mr. Thomas’ classification as a janitor, versus Surgical Assistant to Dr. Blalock, and the absence of support of Mr. Thomas from the larger medical community. Blalock’s lack of mentorship for Mr. Thomas evoked several reactions from the trainees ranging from resignation to sadness to resentment.

Statements 3 through 7 centered on bias recognition. While 80% of the trainees at the beginning of the program answered affirmatively that they could recognize unconscious bias and stereotypes in general (Fig. 1C) as well as in biomedical research, education, and training (Fig. 1D), more than half moved to even greater agreement by the end of the program. With respect to having observed bias in their interactions with their mentors and teachers, most trainees (60%) were neutral initially (Fig. 1E). Half disagreed that they had observed bias in their mentees and another 40% were neutral (Fig. 1G). Only a few (30%) changed their responses to these statements by the end of the program. When it came to observing bias in their peers, half of the trainees were neutral or disagreed at the beginning of the program (Fig. 1F). However, half changed their minds by the end of the program with a mix toward greater or lesser agreement. These changes in response regarding bias in their peers suggest the program increased their understanding of how bias can be manifested, which caused them to re-evaluate the criteria they applied to their colleagues.

In addition to the Movie and Journal Clubs and RCR Series, we invited Dr. Lisa Harrison-Bernard to give two workshops modeled after her professional development course for the faculty at Louisiana State University on *Diversity, Equity, Inclusion and Implicit Bias* (Table 5). Using pre- and post-survey analysis, she showed how her course increased faculty understanding of “implicit bias,” “status leveling,” “color-blind racial attitudes,” “tokenism,” and “failure to differentiate” [19]. The faculty participants also reported increases in their ability “to recognize biases and stereotypes in graduate education, knowing what to say when interacting with people from different cultures, and the ability to acknowledge bias when mentoring students from groups underrepresented in the biomedical field” [19]. We believe her workshops reengineered toward the predoctoral students and postdoctoral fellows in the TBS program contributed to their understanding of conscious and unconscious bias.

At the beginning of the program, 60% of the trainees were affirmative regarding their ability to identify individuals who were underrepresented in science. This moved up to 70% by the end of the program (Fig. 1I). In contrast, 70% disagreed or were neutral regarding their awareness of obstacles faced by these individuals (Fig. 1H). With an increase of 0.9 between average pre- and post-survey scores on the Likert scale, this latter statement revealed the greatest magnitude of positive change in views after implementing our curriculum (Fig. 1H).

Several components of our educational initiative could have contributed to this change. As part of the Visiting Scientist series (Table 5), KAB held a workshop on how perceived racism adversely impacts physiology. The presentation by KAB’s studies suggested that racism contributed to disparities in cardiovascular health [20,21]. She also shared her own research demonstrating that perseverative thoughts associated by negative emotional reactions to perceived racism increase nocturnal autonomic nervous system activity, a potential contributor to the earlier onset of cardiovascular disease in Black populations [22]. This workshop raised awareness of how factors specific to minorities need be considered when studying mechanisms of pathophysiology. The Journal Club focusing on racial and ethnic disparities in COVID-19 outcomes (Table 3) also reinforced this concept as did the RCR training on *Informed Consent and Respect for Personhood* led by RCS (Table 4). In that workshop, RCS raised several examples of egregious racist behavior on the part of the medical community.

The conversation surrounding the film *Miss Evers’ Boys* in the Movie Club was another example. This 1997 docudrama is based upon the Tuskegee study of untreated syphilis that began in 1929 and ended in 1972. The movie focused on Nurse Eunice Evers interactions with the participants who became the victims of the study. The drama describes Nurse Evers facilitation of a program initially intended to treat syphilis among African Americans in rural Alabama. However, over time, it became an observational study in which patients were actively denied treatment, especially after penicillin became available in the 1940s.

The trainees were disgusted by the purposeful withholding of treatment. A lively discussion ensued over Nurse Evers personal conflict, knowingly participating in an unethical study, her role and responsibilities, as well as alternative choices she might have made. There was a lengthy conversation whether the research findings should be published, and if so, with what caveats attached. Some trainees argued the research findings should be published because it honored the sacrifices these men made; however, the researchers’ names should be omitted due to the cruelty of the study. All the trainees agreed treatment should have been given as soon as it was available, despite ending the study prematurely.

Statement 11 referred to seeking out different perspectives from diverse audiences in one’s research. At the beginning of the program, 80% disagreed or were neutral toward this statement, suggesting they did not seek out different perspectives (Fig. 1K). By the end of the program, there was a 0.9 positive change in the average Likert score. Seventy percent changed their response to greater agreement while no one moved to lesser agreement. This response suggesting the program positively impacted trainee behavior is interesting since 90% of the trainees strongly agreed at the beginning of the program that scientists from different cultures improve biomedical research (Fig. 1J). Apparently, the trainees now viewed different viewpoints to be so valuable to one’s research efforts to be worth seeking out. It is likely that the fruitful conversations shared within a safe space throughout the Movie and Journal Clubs, RCR, and Visiting Scientist Workshops may well have contributed to this change in response.

## Discussion

Other NCATS-funded institutions are addressing systemic racism in biomedical research through various platforms. The Center for Leading Innovation and Collaboration posts events offered to the greater Clinical and Translational Science Awards (CTSA) community. Several events have focused on diversity and inclusion (Table 7). It is not known how many training programs require their trainees to attend similar workshops and how many hours of engagement are typical within the training period. It is also not known to what extent such programs change behavior, and if so, to what extent these changes can diminish systemic and structural racism within the wider biomedical research community.

**Table 7.** CTSA sponsored events on systemic racism awareness

Institution/Date	Forum/Title
Pennsylvania State University CTSI	Webinar
October 22, 2020	*Cultivating a Climate of Diversity, Equity, and Inclusion in Virtual Teams* Susan Mohammed, PhD
GHUCCTS	Webinar
October 29, 2020	*Engaging and Integrating Diverse Populations in Research* *GHUCCTS Community Advisory Board*
University of Rochester CTSI	Webinar Series
November 11, 2020	*Science of Dissemination & Implementation, and its Adaptations in Public Health Emergencies* Reza Yousefi Nooraie PhD, MD
November 18, 2020	*Engaging and Linking Stakeholders Across Systems for Implementation* Alicia Bunger MS, PhD Ohio State University
November 25, 2020	*Promoting Health Equity using Dissemination and Implementation: Opportunities and Challenges* Kevin Fiscella MD, MPH
University of Florida CTSI	Black Voices in Research Story Telling Series
January 14, 2021	Tiffany Danielle Chisholm Pineda
June 16, 2021	Erika Moore, PhD Shantrel Canidate, PhD Erica Guerrido, MPH, CPH Samuel Inkabi Tiffany Danielle Chisholm Pineda
June 19, 2021	E. Stanley Richardson ARTSPEAKS Gainesville; Joseph A. Tyndall, MD, MPH Brittany Southern, DVM Joseph A. Tyndall, MD, MPH
Georgia Clinical Translational Science Alliance	Film
February 24, 2021	*Black Men in White Coats*
University of Miami CTSI	Panel Discussion
July 8, 2021	*Recruitment & Retention Strategies for Underrepresented Minorities in Research* Victoria Behar-Zusman, PhD Frank Penedo, PhD Margaret Pericak-Vance, PhD Olveen Carrasquillo, MD, MPH

NCATS-funded institutions addressing systemic racism in biomedical research. CTSI, Clinical Translational Science Institute and the Center for Leading Innovation and Collaboration (CLIC) posts events offered to the greater CTSA community. Several events have focused on diversity and inclusion.

In a recent publication, former NIH director Francis Collins et al. [12] refer broadly to the metrics of success for newly instituted initiatives focusing on structural racism in biomedical science, manifested by health disparities and the biomedical workforce. These include reducing health disparities and inequalities through effective interventions, including increasing the number and funding success rate of NIH grant applications by African Americans, Blacks and Native Hawaiian, and Pacific Islanders. Establishing metrics of success will be essential to determining the efficacy of these NIH strategies to eliminate systemic and structural racism. Likewise, evaluation of the lasting impact of educational initiatives designed to combat systemic racism on future trainee motivation and behavior is critical. To accomplish the global metrics espoused by Collins et al., future studies of a single curriculum implemented at multiple TL1 would enable more rigorous analysis of the effectiveness of training strategies suggested by a single cohort at a single institution. Similar data from other CTS training programs would complement the lessons learned across the national TL1 consortium that could be used to improve graduate and fellowship training programs on addressing systemic racism in general.

### Limitations

The evaluation of our program consisted of a 5.0 Likert scale analysis, which measures perceptions, values, and behavioral changes. However, the scale is not able to capture the richness of our discussions. Another limitation was the small sample size of our TL-1 cohort, which prevented robust statistical analyses and conclusions. Furthermore, our cohort may not be representative of prior and future trainees. We also are not evaluating the lasting impact on future trainee motivation and behavior. Thus, it would be interesting to assess the effectiveness of this educational initiative across diverse training programs in CTS over time and during different social periods. Another limitation is the lack of a “control” group, that is, a similar cohort training in CTS that is not exposed to this educational initiative. This is especially relevant since the TBS program is intentionally diverse in terms of fields of CTS, levels of training, institutions, and departments. Thus, weekly exposure to a diverse cohort of peers and near peers may also have impacted the pre- and post-survey responses.

## Conclusions

The major aspect of our pilot curriculum addressing systemic and structural racism was threading this theme throughout the year-long TBS program. Pre- and post-surveys of our TL1 cohort suggest this integration is an effective educational strategy for early career trainees in CTS. Although the surveys and participation for this pilot program were optional, we had 100% participation. Unlike a stand-alone workshop, this integration enabled us to cover more material on how racism contributes to the pathophysiology of disease, adversely affecting health care, health policy, health outcomes as well as the biomedical scientist while at the same time, providing training in critical competencies of CTS. Incorporating this theme into the curriculum enabled reiteration of themes, a known strategy for reinforcing educational concepts. Furthermore, our emphasis on establishing a safe environment for sharing thoughts and experiences about racial and ethnic challenges, including those from individuals whose ethnicity is underrepresented in science, facilitated the experience. Empowering the trainees to lead and galvanize group conversations around cultural and racial disparities in human health, access to, and use of healthcare services was also instrumental to increasing trainee awareness of these disparities. This enhanced recognition of the negative impact of systemic and structural racism will likely shape their outlook as they pursue their careers. These workshops not only broadened their awareness of racial biases, it also equipped the trainees to approach, support, and advance the conversation to ultimately combat racial and ethnic inequalities in human health and break down systemic institutional barriers. Our program supports the goal of NCATS and other stakeholders to reduce health disparities in CTS. It will be valuable to assess and compare the effectiveness of our program with similar approaches taken by other training programs across the CTSA consortium.
